# Towards Investigating the Effect of Ammonium Nitrate on the Characteristics and Thermal Decomposition Behavior of Energetic Double Base NC/DEGDN Composite

**DOI:** 10.3390/ma15228138

**Published:** 2022-11-16

**Authors:** Hani Boukeciat, Ahmed Fouzi Tarchoun, Djalal Trache, Amir Abdelaziz, Rania Ahmed Hamada, Ayemen Bouhantala, Chamseddine Bousstila, Sabrina Hanafi, Mohammed Dourari, Thomas M. Klapötke

**Affiliations:** 1 Energetic Materials Laboratory (EMLab), Teaching and Research Unit of Energetic Processes, Ecole Militaire Polytechnique, BP 17, Bordj El-Bahri, Algiers 16046, Algeria; 2 Energetic Propulsion Laboratory, Teaching and Research Unit of Energetic Processes, Ecole Militaire Polytechnique, BP 17, Bordj El-Bahri, Algiers 16046, Algeria; 3 Department of Chemistry, Ludwig Maximilian University, Butenandtstrasse 5-13 (D), D-81377 Munich, Germany

**Keywords:** ammonium nitrate, nitrocellulose, diethylene glycol dinitrate, energetic composite, decomposition kinetics

## Abstract

This research work aimed to elaborate on a new modified double-base propellant containing nitrocellulose (NC), ammonium nitrate (AN), and diethylene glycol dinitrate (DEGDN). The developed AN/NC-DEGDN formulation was successfully obtained through a casting process and fully characterized in terms of its chemical structure, morphological features, and thermal behavior. Beforehand, theoretical calculation by the CEA-NASA program was applied to select the optimal composition of the formulation. Experimental findings demonstrated the homogenous dispersion of AN oxidizer in the NC-DEGDN matrix without alteration of their molecular structures. The catalytic influence of AN on the thermal decomposition behavior of NC-DEGDN film was also elucidated by thermal analyses. When AN was incorporated into the formulation, the decomposition peak temperatures for the different decomposition processes were shifted toward lower temperatures, while the total enthalpy of decomposition increased by around 1272.24 J/g. In addition, the kinetics of the thermal decomposition of the developed modified double base propellant were investigated using DSC results coupled with model kinetic approaches. It was found that the addition of AN decreases the activation energy of nitrate esters from 134.5 kJ/mol to 118.84 kJ/mol, providing evidence for its excellent catalytic effect. Overall, this investigation could serve as a reference for developing future generation of modified double-base propellants.

## 1. Introduction

Nowadays, the development and characterization of new energetic formulations have addressed numerous challenges in the field of insensitive munitions and solid rocket propellants [1,2]. As a crucial branch of energetic formulations, modified double-base solid propellants (MDBs), corresponding to energetic composites that contain cellulose nitrate (NC) as a binder, plasticizers, oxidizers (ammonium perchlorate, AP), energetic additives (e.g., nitramines), stabilizers, and catalysts, among others, play a prominent role in chemical propulsion systems [3]. Such energetic formulations are known for their excellent energetic performance, interesting mechanical characteristics, attractive operational features, and mature processing technology, increasing their demand for several military uses in missiles, rocket motors, and boosters [4]. As an important ingredient in MDBs, energetic plasticizers such as nitroglycerine (NG), diethylene glycol dinitrate (DEGDN) and ethylene glycol dinitrate (EGDN) act as a fuel and improve the overall energy level, plasticity, and physical characteristics of the propellant [5,6]. Broadly, an appropriate plasticizer should display acceptable sensitivity, compatibility with other components, suitable melting point, and good density [6]. Nonetheless, nitroglycerine, the most largely employed energetic plasticizer, suffers from various problems, including sensitivity to friction and impact, low oxygen balance (3.5%), reduced thermal stability, and migratory issues [7,8,9]. To address the above-mentioned issues associated with traditional NG reliance, several emergent plasticizers comprising DEGDN and EGDN are revealed as promising candidates to replace NG, owing to their inherent characteristics such as improved participation and wetting features that lead to softer boundaries at NC interfaces, thus decreasing the sensitivity of the energetic formulation. Furthermore, they can decrease the freezing temperature of nitroglycerine [10]. As an energetic plasticizer, EGDN was found to be more effective than NG and present high energy, while being less sensitive to impact [11]. In one research paper, Yunlu Li et al. [6] mentioned that the introduction of hydrogen bond functional groups such as -NH_2_, -CH_3,_ or other functions into energetic compounds would enhance their safety. Therefore, the use of DEGDN, which forms homogenous matrices with NC promoted by the electrostatic N–O interactions and the hydrogen bond network, has actually attracted much attention as a prominent alternative plasticizer in energetic formulations [8,9]. However, such propellant formulations (NC-DEGDN) demonstrate reduced energetic performance due to their low oxygen balance [10]. To address this issue, oxidizers such as ammonium perchlorate (AP), hydrazinium nitroformate (HNF), ammonium nitrate (AN), ammonium dinitramide (ADN), and hexanitrohexaazaisowurtzitane (CL-20) were introduced to the double-base energetic formulation to compensate the oxygen deficiency and allow producing effective propellant composites with better energetic performance [12]. MDBs based on nitrate esters (NC and NG) and AP as an oxidizer have been widely employed for military purposes owing to their high oxygen balance of 34% [12]. Nevertheless, the use of AP causes serious problems of toxicity and corrosiveness due to the released hydrochloric acid (HCl) [12,13]. Therefore, AN is actually used in most composite propellants to replace AP owing to its low cost, its coating, the effect of additives, its applications and usability [12,14,15]. In addition, the good thermal stability of AN and its ability to form homogenous systems with NC and DEGDN motivated us to explore this new composite-modified double-base solid propellant [7,16]. To the best of our knowledge, MDBs based on nitrocellulose, diethylene glycol dinitrate and ammonium nitrate have never been studied. Therefore, investigation of the characteristics and thermal behavior that occur within such energetic composites is crucial for developing future generations of modified double-base propellants.

The main purpose of the present investigation was to elucidate the potential effect of AN on the characteristics and thermo-kinetic decomposition of NC-DEGDN. The energetic composite of AN/NC-DEGDN was firstly optimized using NASA-CEA software, where the specific impulse was calculated. The characteristics of the optimized energetic composite were characterized using, respectively, scanning electron microscopy (SEM), Fourier transform infrared spectroscopy (FTIR), and an electronic densimeter. The thermal features of AN/NC-DEGDN were evaluated by thermogravimetric analysis (TGA) and differential scanning calorimetry (DSC). The kinetic triplet, namely, the activation energy, the pre-exponential factor, and the best reaction model were also predicted based on isoconversional kinetic methods.

## 2. Experimental Section

### 2.1. Materials

AN powder used in the present research was commercial grade purchased from Prolabo. Cellulose nitrate (NC) with a nitrogen content of 12.68% was prepared following the method detailed in our recent studies [17,18]. All other reactants and solvents were of analytical grade and were used without further purification.

### 2.2. Preparation of DEGDN Plasticizer

The preparation method for DEGDN was inspired by the study of Yahya et al., with some modifications [9]. Briefly, the sulfonitric acid was prepared by dropwise addition of nitric acid (8 mL, 99%) to sulfuric acid (8 mL, 98%). The obtained acid mixture was cooled to 5 °C, and a certain amount of dichloromethane (60 mL) was added. After that, diethylene glycol (DEG) (0.04 mol) was slowly poured, under stirring, into the solution. The mixture was then stirred for 35 min. Next, the bottom acidic phase was removed, while the organic phase was rinsed, respectively, with water, sodium bicarbonate, 10% urea, and sodium chloride. It is important to mention that after each stage of purification, our solution was shaken, and only the lower phase was recuperated. Lastly, the resulting product was filtered using a rotary evaporator (Laborota 4000, Heidolph, Schwabach, Germany). The purity of the obtained DEGDN was 99.9%.

### 2.3. Evaluation of the Theoretical Performance of CSPs

Before proceeding to the formulation of the energetic composite, an evaluation of the theoretical performance was performed using NASA Chemical Equilibrium with Applications (CEA) thermochemical software in order to select the ideal composition [19]. It is interesting to mention that Bondarchuk recently developed a new approach to perform a preliminary crude estimation of the detonation performance of a C-H-N-O energetic material without quantum-chemical calculations [20]. The proposed method can be also effectively used to design new energetic materials or modify existing ones.

**Figure 1:**
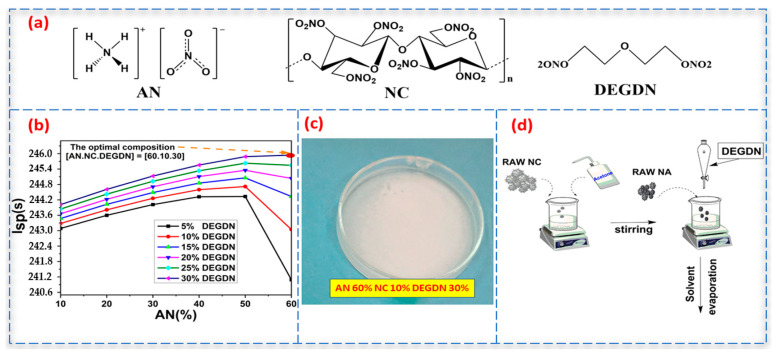
(**a**) Chemical structures of AN, NC, and DEGDN. (**b**) Variation of the specific impulse of AN/NC-DEGDN formulation versus the mass fraction of AN. (**c**) AN/NC-DEGDN film. (**d**) Elaboration process of AN/NC-DEGDN formulation.

In the case of solid rocket propellants, specific impulse is considered a crucial energetic performance parameter to measure the efficiency of a rocket motor. Therefore, this parameter, which can reach high values when the ratio between flame temperature and the combustion product’s molar mass increases, is used as a selection criterion. The MDBP composition was optimized and simulated with AN as an oxidizer and NC/DEGDN as a matrix. It is worth noting that the percentage of DEGDN used in this research ranged from 5% to 30% in order to put the system on a scale and select the maximal specific impulse. The chemical structures of the studied samples are shown in Figure 1a, whereas the obtained findings corresponding to the calculated specific impulse (I_sp_) are illustrated in Figure 1b.

### 2.4. Elaboration of AN/NC-DEGDN Propellant

The dried nitrocellulose was firstly solubilized in acetone for 30 min at room temperature. After that, DEGDN was introduced to the mixture under stirring for 15 min. Then, AN was slowly included in the NC-DEGDN mixture. Next, the mixture was placed in a Petri dish to allow the solvent’s evaporation and dried at 60 °C. The weight fractions of the compounds used to prepare our MDBP, which represented the optimal composition calculated using the CEA program, were equal to 60% AN, 10% NC, and 30% DEGDN. The elaboration pathway is presented in Figure 1d, while a digital image of the as-prepared MDBP is depicted in Figure 1c.

### 2.5. Characterization Methods

A scanning electron microscope (SEM) apparatus (model FEI Quanta 60, Hillsboro, Oregon, USA), through a secondary electron detector at an accelerating voltage of 5 kV, was used to examine the surface morphology and the homogeneity of the different samples. A Perkin Elmer spectrometer (Waltham, Massachusetts, USA) was used to study the chemical structure using Fourier transform infrared spectroscopy (FTIR) at ambient temperature in the wavenumber range of 4000 to 400 cm^−1^. All FTIR spectra were captured in ATR mode with an accumulation of 64 scans and a resolution of 4 cm^−1^. Thermal characteristics of the investigated samples were elucidated by Perkin-Elmer TGA 8000 analyzers (Waltham, Massachusetts, USA). After sample drying, a sample mass of 0.8–1 mg was analyzed in the temperature range of 50 °C to 500 °C at a heating rate of 10 °C/min. All measurements were recorded under a constant nitrogen atmosphere (30 mL/min) to prevent oxidation effects. In order to evaluate the thermo-kinetic factors of the developed MDBP, differential scanning calorimetry (DSC) measurements were performed on a Perkin-Elmer DSC 8000 analyzer (Waltham, Massachusetts, USA) for approximately 1 mg specimens at different heating rates (5, 10, 15, and 20 °C/min) under nitrogen atmosphere from 50 °C to 350 °C.

### 2.6. Kinetic Modeling

A kinetic evaluation was carried out by applying isoconversional kinetic analysis to the non-isothermal DSC data in order to better understand the thermal behavior of the developed AN/NC-DEGDN propellant. According to the recommendations of the International Confederation for Thermal Analysis and Calorimetry (ICTAC), at constant conversion (α), the only factor affecting reaction rate is temperature. Basically, the progress of the conversion versus time is given by Equation (1).

(1) $$\frac{d\alpha }{dT}=k\left(T\right)f\left(\alpha \right)\;$$

where *T* refers to the temperature at *t* instant, *k (T)* presents the rate constant, and *f*(α) stands for the differential model. The value of α (0< α <1) can be derived from the peak area of the DSC curves, as the ratio of the current enthalpy ΔH to the total enthalpy $\Delta H{\;}_{total}$, as shown in Equation (2).

(2) α=∫t0t(dHdt)dt∫t0tf(dHdt)dt=ΔH ΔH total

Two isoconversional linear methods, called Trache-Abdelaziz-Siwani (TAS) [21], iterative Kissinger-Akahira-Sunose (it-KAS) [22], and Vyazovkin’s non-linear approach combined with the compensation effect method (VYA/CE) [23], were employed to predict the Arrhenius parameters (the activation energy (*E_a_*) and the preexponential factor (*Log* (*A*))) and the most probable mechanism of decomposition (*g*(*α*)).

## 3. Results and Discussions

### 3.1. Evaluation of the Optimal Composition

The theoretical investigation of the energetic properties is important to choose the optimal composition of propellant formulations with the best performance. The main purpose of the current investigation was to determine the impact of AN on the specific impulse (*Isp*) of the NC/DEGDN formulation. A tertiary variation between the basic constituents, namely DEGDN (5% to 30%) and AN (10% to 60%), was studied. For practical feasibility, a minimum and maximum amount of 5% and 30%, respectively, were considered for DEGDN. It can be revealed from Figure 1b that by varying the weight fraction of the AN/NC-DEGDN compounds within the composite, the values of *Isp* were determined. It was found that the increase in AN content improved the *Isp*, which was explained by the rise in oxygen content and the gaseous decomposition of AN [14]. This growth in oxygen balance could also improve the flame temperature [24]. It was also found that the insertion of AN into NC/DEGDN increased the flame temperature from 2625 K for NC/DEGDN (1:3 wt.%) to 2716 K for the optimal AN/NC-DEGDN (60:10:30, wt.%) formulation. This augmentation in the flame temperature promoted the *Isp* from 244.6 s to 245.9 s. This result suggests that raising the flame temperature can improve the AN/NC-DEGDN formulation’s burning rate, which can be also enhanced by the exothermic processes that happen within the propellant, as demonstrated by DSC. Additionally, it is worth mentioning that the AN content within the prepared MDBP was less than that employed in the standard composite propellants, which broadly ranged between 65–80% [25,26].

### 3.2. Morphology and Chemical Structure

**Figure 2:**
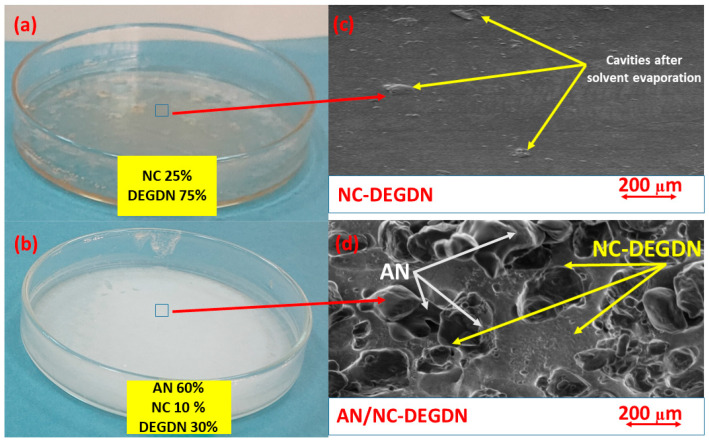
(**a**) NC-DEGDN composite film; (**b**) AN/NC-DEGDN composite film; SEM images of ((**c**) NC-DEGDN mixture, (**d**) AN/NC-DEGDN mixture).

**Figure 3:**
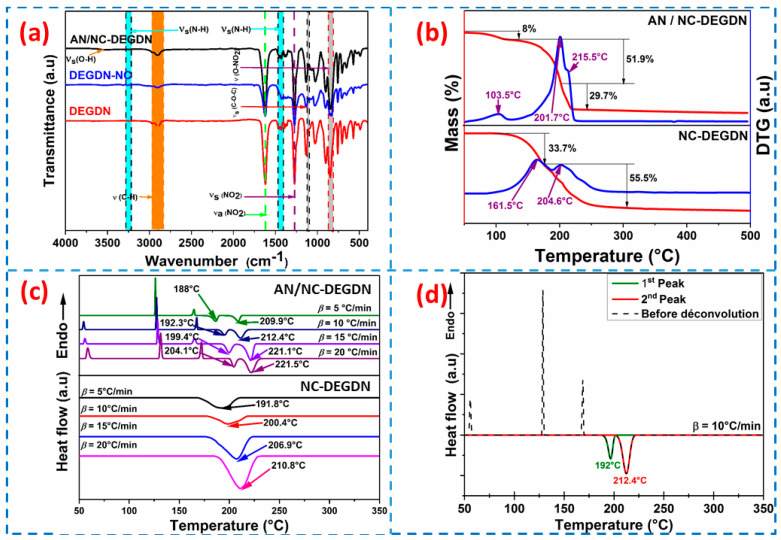
(**a**) FTIR spectra of DEGDN, NC/DEGDN, and AN/NC-DEGDN. (**b**) TG-DTG profiles of mixtures at 10 °C/min. (**c**) DSC thermograms of the different samples at different heating rates of 5, 10, 15 and 20 °C/min. (**d**) Mathematical deconvolution of the DSC thermogram of AN/NC-DEGDN formulation at *β* = 10 °C/min.

The morphological features and the chemical structure of the as-prepared MDBP were assessed by scanning electron microscopy and infrared spectroscopy, respectively, and the obtained findings are illustrated in Figure 2 and Figure 3. It is clear from the first screening of the digital images shown in Figure 2a,b that homogeneous and uniform structures were formed either with or without the presence of AN.

A comparison between the morphologies of the NC-DEGDN and AN/NC-DEGDN composites is shown in Figure 2c,d. As can be seen, the double-base film without AN exhibited a uniform structure with a smooth surface, which was referred to as good dissolution of NC/DEGDN. Furthermore, the observed sheet structure after the dissolution pathway was improved by the addition of DEGDN. However, a few holes on the sheet surface could be detected, which was probably caused by small solvent droplets evaporating. Additionally, a few NC aggregates were visible, which was probably due to the intermolecular interactions between the NC fibers. Figure 2c,d shows the difference in morphology after the introduction of AN. It was found that when AN microparticles were added, the smooth surface disappeared, which was evenly distributed throughout the NC-DEGDN film or surface-coated in. Such efficient dispersion can improve the interfacial interactions among the compounds and is expected to promote the decomposition temperatures of the energetic formulation.

To appreciate what was previously stated, an important parameter called density must be measured. According to the current trend in energetic materials, high densities lead to better energy performance and a high burning rate. For each sample, to assess their experimental densities, 10 analyses were carried out to determine the average density and standard deviation with an electronic Accupyc 1340 II densimeter. It was revealed that the presence of AN within the NC-DEGDN film provided an increased density of 1.5872 ± 0.0013 g/cm^3^ with respect to the NC-DEGDN formulation, which presented a value of 1.4271 ± 0.0054 g/cm^3^. This increase in density is expected to enhance the performance of the developed MDBP. In addition, the obtained experimental densities were found to be comparable to their theoretical densities, corresponding to 1.6178 g/cm^3^ for AN/NC-DEGDN and 1.4570 g/cm^3^ for NC-DEGDN, thus implying that the open porosity of the designed energetic composites was very low and demonstrating the high loading capacity of the NC-DEGDN formulation. These outcomes agreed well with those obtained by SEM, and highlight the potential advantages of adding AN to develop a promising energetic formulation for advanced applications.

FTIR characterization was carried out to elucidate the chemical structure of the developed energetic composites, and the obtained spectra are shown in Figure 3a. It is clear that both composites exhibited a large absorption peak at 2990~2850 cm^−1^, which corresponded to the stretching vibration of C-H available in NC and DEGDN [27]. The other characteristic bands, observed at 1620 cm^−1^, 1270 cm^−1^, and 814 cm^−1^, were attributed to the asymmetric stretching of the N–O bond in NO_2_, the symmetric elongation of -NO_2_ and the twisting vibration of O–NO_2_, respectively [9]. In addition, the absorption band, located at 1114 cm^−1^, was assigned to the overlapped vibration of C-O-C [28]. The other bands associated with NC and/or DEGDN were comparable to those mentioned in previous works [19,29,30]. Additional peaks that could be observed when AN was introduced to the NC-DEGDN formulation were related to the characteristic functional groups of this oxidizer. For instance, the bands detected at 3260 cm^−1^ and 1417 cm^−1^ were attributed, respectively, to the stretching and bending vibrations of N-H. In addition, the presence of a typical vibrational peak of -O-H at 3500 cm^−1^ confirmed the hydrogen bond interactions between AN and NC-DEGDN [31]. Besides that, there were no alterations in the chemical structures of AN or NC-DEGDN as originated by the preparation pathway, providing evidence for good chemical compatibility between the different energetic compounds and confirming the successful elaboration of the desired composites.

### 3.3. TGA Characterization

The thermal decomposition features of the developed MDBP formulations were investigated by TGA, and the obtained TGA/DTG thermograms are plotted in Figure 3b. As can be seen, NC-DEGDN composite showed two weight-loss events appearing at DTG peak temperatures of 161.5 °C and 204.6 °C with a weight loss of 34% and 56%, respectively. The first mass loss was mainly induced by the volatilization and decomposition of DEGDN [28,32], whereas the second stage of decomposition was attributed to the well-known thermolysis process of nitrocellulose [33]. After the addition of AN, it was evident, as seen in Figure 3b, that the obtained AN/NC-DEGDN composite exhibited three decomposition stages. The first step, at 103.5 °C, was mainly caused by water evaporation. The second and the last weight losses, observed at 201.7 °C and 215.5 °C, were related to the decomposition of NC-DEGDN matrix and AN, respectively [34,35]. It is important to note that on reaching 500 °C, both energetic formulations (NC/DEGDN and AN/NC-DEGDN) lost around 90% of their initial weight. In addition, the influence of AN on the thermolysis of the NC-DEGDN composite, as clearly revealed from the presented TGA/DTG thermograms, notably decreased the decomposition temperatures of the obtained AN/NC-DEGDN propellant. Therefore, the first event of NC-DEGDN decomposition was accelerated by the highly reactive products generated during the decomposition of AN oxidizer. Such behavior was also reported by Abd-Elghany et al. [36], who highlighted that the addition of an oxidizer into an NC-based formulation accelerates the thermolysis and the combustion performance. Another interesting finding was that the thermal decomposition of AN was also shifted to a lower temperature (from 260 °C for pure AN to 215.5 °C)**,** which was probably due to the early thermolysis of NC-DEGDN that catalyzed the decomposition of AN. According to the above discussion, we can conclude that the thermal behavior of the developed MDBP was dominated by the decomposition of the NC-DEGDN composite.

### 3.4. DSC Characterization

To carefully investigate the influence of AN on the thermal reactivity and enthalpy of the decomposition of NC-DEGDN propellant and elucidate the thermolysis events of the developed AN/NC-DEGDN formulation, DSC characterizations were also carried out at four heating rates to identify the exothermic/endothermic decomposition events and to calculate the kinetic parameters of thermal decomposition.

The obtained DSC curves of NC/DEGDN with or without AN composites at various *β* are depicted in Figure 3c, while the onset and maximum peak temperatures (*T*_onset_ and *T*_peak_), as well as the enthalpy of reaction (∆*H*) at *β* = 10 °C/min, are listed in Table 1. Based on Figure 3c, the NC-DEGDN mixture displayed one exothermic process, comparable to what is mainly obtained for double-base propellants [17,37]. However, two exothermic events were detected for the AN/NC-DEGDN formulation, comprising the decomposition peaks of NC-DEGDN matrix and AN oxidizer [34,35]. Furthermore, the two first endothermic processes, which were independent of the heating rate, corresponded, respectively, to two solid–solid transitions. The first one, which appeared at around 50 °C, was attributed to orthorhombic–orthorhombic transition, where a modification of crystalline parameters occurred, while the second one, observed at around 123 °C, was due to the tetragonal–cubic transition. These processes were then followed by a melting phenomenon of AN [12]. It is worth mentioning that the first peak encountered in the TGA curves of the investigated energetic composites, attributed to the evaporation process, did not appear in the DSC result, which was due to the lower mass employed in the DSC, and that the amount of absorbed energy during the endothermic evaporation process was beyond the lower limits of the DSC device.

Additionally, it is clear from Table 1 and Figure 3c that the addition of AN into NC-DEGDN significantly affected the thermolysis of the NC-DEGDN matrix, where its decomposition peak moved to lower temperature, taking advantage of the excellent interfacial contact between NC-DEGDN matrix and AN particles, as demonstrated by SEM. This behavior could be related to the nitrate ions in AN that improve the heat and mass transfer within the nitrate esters and accelerate their thermolysis, which could also be sustained by the difference between the peak and the onset temperatures (∆*T*) for AN/NC-DEGDN, which notably decreased with regard to those of the NC-DEGDN baseline (Table 1). In addition, it was found that the overall enthalpy of decomposition of AN/NC-DEGDN (2896.84 J/g) appeared to be higher than that of the NC-DEGDN matrix (1624.6 J/g). This finding seems to be promising for future propellant formulation and indicates the positive effects of AN on the increase in the energetic performance of the developed energetic formulation. Additionally, it is worth noting that the newly designed AN/NC-DEGDN propellant has better or comparable thermal stability than some reported NC-based energetic formulations, including dihydroxylammonium 5,5′-bistetrazole-1,1′-diolate/NC-NG (*T*_peak_ = 170.9 °C, *β* = 10 °C/min), hexahydro-1,3,5-trinitro-1,3,5-triazine/NC-TEGDN (*T*_peak_ = 194.9 °C, *β* = 10 °C/min) and NC/HMX (*T*_peak_ = 168.2 °C, *β* = 10 °C/min) [38].

### 3.5. Evaluation of Thermal Decomposition Kinetics

Owing to the risk of thermal runaway in NC-based composites, it is crucial to analyze their thermal behaviors. Therefore, the DSC data obtained at various heating rates (i.e., 5, 10, 15 and 20 °C/min) were used to determine the kinetic triplet, namely, the activation energy (*Ea*), the pre-exponential factor (*Log*(*A*))*,* and the most probable reaction model (*g*(*α*)). Before examining the computed kinetic factors, it is interesting to define them. *Ea* represents the lower energy needed to initiate a reaction, whereas the collision rate between molecules per unit of time is determined by *Log*(*A*). Most of the time, *Log*(*A*) may be computed using a model-free technique called compensating effect, which requires that *Ea* and *Log*(*A*) have a linear relationship [18,39].

As advised by the ICTAC, the asymmetric Frazer–Suzuki function, the most popular technique for accurately fitting the multistep kinetic decomposition, was used for peak deconvolution of the DSC thermogram of the AN/NC-DEGDN formulation. The obtained data were then subjected to three model-free methods to determine the kinetic parameters for each exothermic decomposition event. The mean values of *E_a_* and *Log*(*A*) with their corresponding errors for both energetic composites (NC/DEDGN and AN/NC-DEDGN), as well as their most probable mechanisms g(α), are given in Table 2.

The main result was that the computed *E_a_* and *Log(A)* using the three isoconversional models for both propellants (NC/DEDGN and AN/NC-DEDGN) agreed well with each other, highlighting the high consistency of the computed calculations. The accurateness of the determined Arrhenius factors using the linear TAS and it-KAS approaches also could be demonstrated by the high value of the linear correlation coefficients (*R^2^*) higher than 0.9993 [40,41,42,43].

**Figure 4:**
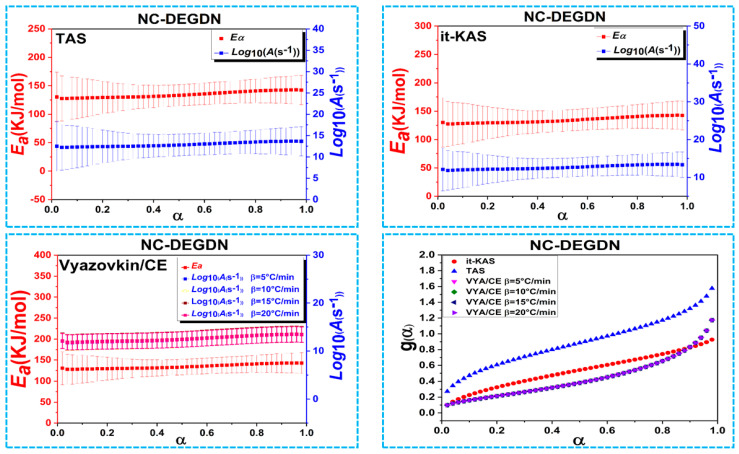
Variation of the kinetic parameters versus conversion for the thermal decomposition of NC-DEGDN composite.

**Figure 5:**
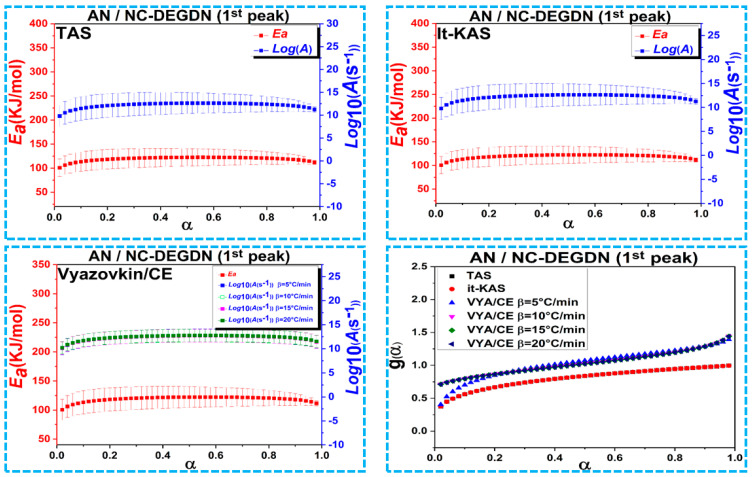
Variation of the kinetic parameters versus conversion for the first thermal decomposition event of AN/NC-DEGDN propellant.

**Figure 6:**
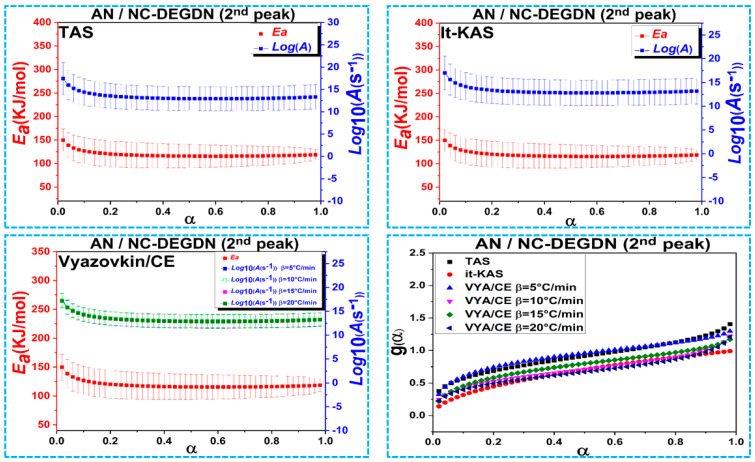
Variation of the kinetic parameters versus conversion for the second thermal decomposition event of AN/NC-DEGDN propellant.

Figure 4, Figure 5 and Figure 6 represent the evolution of activation energy and the pre-exponential factor, with their associated confidence intervals, and the most probable reaction model g(α) as a function of conversion (α) for the thermolysis process of NC/DEDGN, AN/NC-DEDGN 1st stage, and AN/NC-DEDGN 2st stage, respectively. In order to avoid the inherent errors related to the initial and end periods, the conversion *α* range was fixed from 0.02 to 0.98. The resulting findings show that the employed kinetic approaches provided close values and similar trends of Arrhenius parameters for each investigated sample. Another finding was that the evolution trends of *Ea* and *Log(A)* with conversion, for each decomposition event, were identical, which was explained by the energy compensation effects [44].

It can be clearly seen in Figure 4 and Table 2 that the decomposition of double-base NC/DEDGN propellant was controlled by a one-step process described by a stable evolution of Arrhenius parameters [42]. Furthermore, it is worth noting that the *E*_a_ of NC-DEGDN predicted in this study (134.5 ± 24 kJ/mol) agreed well with that found for other double-base propellants [9]. The incorporation of AN into the NC-DEGDN film led to a decrease in *E*_a_ values by about 12% (Figure 5), which was attributed to the catalytic effect of AN that accelerated the thermolysis process and boosted both heat and mass transfers within the nitrate esters. The same behavior was mentioned in the studies of Jain et al. and Li et al., who reported an improvement in the thermolysis rate of nitrate esters when AP was introduced [45,46]. Moreover, the average value of *E*_a_ (≈ 119 kJ/mol) for the second decomposition event, which corresponded to the decomposition of AN in NC/DEGDN matrix, was found to be lower than that of pure AN reported in previous work (*E*_a_ ≈ 146 kJ/mol) [44,47].

Other important parameters to determine in the kinetic analysis of the prepared energetic formulations were their most probable mechanisms (g(*α*)) derived from the used kinetic approaches. The mathematical formula of g(*α*) is summarized in Table 2, and its evolution vs. α is illustrated in Figure 4, Figure 5 and Figure 6. It is interesting to note that the Vyazovkin approach did not provide a mathematical formula of g(*α*), but its combination with the compensation effect could offer numerical values of the model. According to the obtained results, we could conclude that the studied samples decomposed following different mechanisms. Referring to the TAS method, the NC-DEGDN composite decomposed following the random nucleation process of Avrami–Erofeev. However, the addition of AN to the NC-DEGDN, which caused a drop in *E*_a_, changed the decomposition behavior of the nitrate esters in NC/DEGDN from A_5/2_ to P_1/4_, which conformed to the nucleation process (power law) for both the TAS and it-KAS methods. However, the second stage of decomposition, which corresponded to the exothermic decomposition of AN, followed an Avrami–Erofeev mechanism (A_4_) [34]. On the other hand, the same model was provided by it-KAS for the 1st and 2nd stages of AN/NC-DEGDN, whereas NC-DEGDN decomposed following the G7 model. Overall, the findings of this study give new insights into the importance of developing energetic double-base composites based on DEGDN and AN rather than the use of highly sensitive NG [48,49].

## 4. Conclusions and Future Work

A new, modified double-base solid propellant (AN/NC-DEGDN) was successfully prepared through AN dispersion in a mixture of NC and DEGDN. The optimal formulation, which corresponded to AN/NC-DEGDN (60/10/30 wt.%), was obtained based on maximizing the critical parameter, the specific impulse, using the CEA-NASA program. Morphology characterization and density assessments of the prepared energetic composites indicated an appropriate homogeneity of AN in the NC-DEGDN formulation with good dispersion. The AN/NC-DEGDN composite showed lower thermal and kinetic characteristics than the NC-DEGDN composite, while a higher enthalpy of decomposition was obtained, demonstrating the positive catalytic influence of the incorporation of AN. Furthermore, the thermolysis of AN was also increased by the released hot reactive species, which was expected to enhance the burning rate of the propellant. In addition, the employed isoconversional kinetic models revealed that the developed energetic formulations decomposed following different mechanisms, which could change from a random nucleation mechanism of Avrami–Erofeev to power law nucleation. Overall, it can be concluded that the developed AN/NC-DEGDN formulation, which has inherent thermal and kinetic characteristics as well as high energetic performance with respect to the NC-DEGDN baseline, can be seen as a promising candidate for advanced high-performance modified double-base propellants in the future.

## Figures and Tables

**Table 1:** Thermal characteristics of the investigated energetic composites obtained at a heating rate of *β* = 10 °C/min.

Sample	1st Decomposition Stage				2nd Decomposition Stage				
	*T*_onset_ (°C)	*T*_peak_ (°C)	∆*T* * (°C)	∆*H* (J/g)	*T*_onset_ (°C)	*T*_peak_ (°C)	∆*T* * (°C)	∆*H* (J/g)	∆*H_T_* (J/g)
NC-DEGDN	182.5	200.4	17.9	1624.6	/	/	/	/	1624.6
AN/NC-DEGDN	187.5	192.3	4.8	1961.8	204.4	212.4	8	935.04	2896.84
AN	/	/	/	/	300.04	318.2	24	805	803–805 [35]

* ∆*T* =*T*_peak_ − *T*_onset_; ∆*H_T_*, total heat release.

**Table 2:** Thermo-kinetic parameters of the elaborated energetic formulations.

Samples	Isoconversional Methods		*Eα* (kJ/mol)	*Log*(*A*(*s*^−1^))	*g*(*α*)
AN/NC-DEGDN 1st Pic	TAS		118.9 ± 16.6	12.2 ± 2.1	P_1/4_ = a^1/4^
	it-KAS		118.8 ± 16.5	12.2 ± 2.0	P_1/4_ = a^1/4^
	VYA/CE	*β* = 5 °C/min	118.8 ± 16.4	12.3 ± 1.4	/
		*β* = 10 °C/min		12.6 ± 1.4	/
		*β* = 15 °C/min		12.7 ± 1.4	/
		*β* = 20 °C/min		13.8 ± 1.4	/
AN/NC-DEGDN 2nd pic	TAS		119.2 ± 23.3	13.4 ± 2.8	A_4_ = [− ln (1 − *α*)]^1/4^
	it-KAS		119.2 ± 23.3	13.2 ± 2.8	P_1/2_ = a^1/2^
	VYA/CE	*β* = 5 °C/min	119.1 ± 20.9	13.3 ± 1.4	/
		*β* = 10 °C/min		13.4 ± 1.4	/
		*β* = 15 °C/min		13.3 ± 1.4	/
		*β* = 20 °C/min		13.3 ± 1.4	/
NC-DEGDN	TAS		134.5 ± 23.9	12.9 ± 3.2	A_5/2_ = [− ln (1 − *α*)]^2/5^
	it-KAS		134.5 ± 23.9	12.7 ± 3.2	G_7_ = [1 − (1 − *α*)^1/2^]^1/2^
	VYA/CE	*β* = 5 °C/min	134.4 ± 23.9	13.3 ± 3.3	/
		*β* = 10 °C/min		13.4 ± 3.3	/
		*β* = 15 °C/min		13.5 ± 3.3	/
		*β* = 20 °C/min		13.6 ± 3.3	/

## Data Availability

Not applicable.
